# American Health Association

**Published:** 1887-01

**Authors:** 


### AMERICAN HEALTH ASSOCIATION.

That vigorous organization, known as the American Health Association, held its annual meeting for 1886 in the City of Toronto. This association is composed of all gentlemen who take an interest in sanitary matters, and embraces in its scope the consideration of all subjects bearing on public health, whether belonging to human or veterinary medicine. The prospect of the meeting had excited much interest, and the sanitarians of the province and city welcomed their visitors warmly. The visiting members were from almost every important point on this continent, and came in such numbers as to render this meeting of exceptional interest. The meetings continued from October 5th to the 8th, and were presided over by Dr. H. P. Walcott with rare ability. Among the many valuable papers presented, one by D. E. Salmon, D. V. M., may be mentioned as of special interest in present circumstances, on "Recent Progress in the Investigation of Hog Cholera." Nearly fifty Canadian members were added to the association, while one of its results appears in the establishment of an Association of Health Officers for the Province of Ontario.

